# The association of aspirin use with overall survival of patients with inoperable non-small cell lung cancer: a retrospective study

**DOI:** 10.1186/s12885-021-08999-8

**Published:** 2021-11-22

**Authors:** Min-Chun Chuang, Yao-Hsu Yang, Meng-Jer Hsieh, Yu-Ching Lin, Tsung-Ming Yang, Pau-Chung Chen, Ming-Szu Hung

**Affiliations:** 1 Department of Pulmonary and Critical Care Medicine, Chang-Gung Memorial Hospital at Chiayi, Chang-Gung Medical Foundation, No. 6, West Section, Chia-Pu Road, Pu-Tz City, Chiayi 613 Taiwan; 2 Department of Respiratory Therapy, School of Medicine, Chang-Gung University, Taoyuan City, Taiwan; 3 Department of Traditional Chinese Medicine, Chiayi Chang Gung Memorial Hospital, Chiayi, Taiwan; 4 Health Information and Epidemiology Laboratory of Chang Gung Memorial Hospital, Chiayi, Taiwan; 5 School of Traditional Chinese Medicine, College of Medicine, Chang Gung University, Taoyuan, Taiwan; 6 Institute of Occupational Medicine and Industrial Hygiene, National Taiwan University College of Public Health, Taipei, Taiwan; 7 Department of Environmental and Occupational Medicine, National Taiwan University Hospital and National Taiwan University College of Medicine, Taipei, Taiwan; 8 Department of Respiratory Care, Chang Gung University of Science and Technology, Chiayi Campus, Chiayi, Taiwan

**Keywords:** Aspirin, Non–small cell lung cancer, Overall survival

## Abstract

**Background:**

Studies have indicated that individuals taking aspirin have a reduced risk of cancers and have also established chemo-preventive benefit of aspirin in colorectal cancer. However, research on the association between aspirin use and the survival in patients with lung cancer has revealed inconsistent results. In this study, we investigated the effect of aspirin use on the survival of inoperable non-small cell lung cancer (NSCLC) patients.

**Methods:**

We identified a cohort of 38,842 patients diagnosed with NSCLC between 2000 and 2012 using the Taiwan’s National Health Insurance Research Database and used propensity score matching to reduce possible confounding factors. In total, 9864 patients (4932 matched pairs) were included in the matched cohort. Aspirin exposure was analyzed to identify a possible association with mortality in patients with inoperable NSCLC. Time-dependent Cox regression models were used to calculate the hazard ratios (HRs) and the 95% confidence intervals (95% CIs) that corresponded with aspirin exposure.

**Results:**

A total of 4979 patients used aspirin at the time of diagnosis of NSCLC. The median overall survival (OS) of the aspirin users was 1.73 (interquartile range, 0.94–3.53) years compared with the 1.30 (interquartile range, 0.69–2.62) years of the non-aspirin users. The Cox proportional hazard model with the time-dependent covariate revealed that aspirin use was associated with a significantly longer OS (HR: 0.83, 95.0% CI: 0.80–0.86). After controlling the sociodemographic characteristics (age, sex, income, and level of urbanization) and lung cancer treatments by propensity score matching, the aspirin users still had a significantly longer OS than the non-aspirin users (HR: 0.79, 95.0% CI: 0.75–0.83).

**Conclusion:**

Aspirin use is associated with a longer OS in patients with inoperable NSCLC, suggesting that aspirin has a potential anticancer effect. These results warrant further randomized clinical trials to evaluate the actual role of aspirin in the treatment of NSCLC patients.

## Background

Despite significant advances in the treatment and diagnosis over the last few decades, lung cancer remains the leading cause of cancer mortality (1.8 million deaths; 18.0% of all cancer deaths) and the second most commonly diagnosed cancer (2.2 million cases; 11.4% of all cancer cases) worldwide in 2020 [1]. In Taiwan, lung cancer has been the top-ranking cause of cancer deaths for many years [2].

Non-small cell lung cancer (NSCLC) accounts for the majority of all lung cancer cases, and approximately 65% of patients present with locally advanced or metastatic disease at the time of diagnosis [3]. Even with the use of aggressive multimodality therapy, the prognosis in patients with advanced stage NSCLC has been far from satisfactory [4]. To better cope with this global health burden, the identification of effective methods to improve the therapeutic efficacy of NSCLC is of prime importance.

Recent studies have demonstrated the activity of old drugs on novel anti-cancer pharmacological targets. Repurposing aspirin as antineoplastic agents has gathered momentum because of its potential anti-cancer effect both in vitro and *in* vivo [5]. The direct inhibition of the activity of cyclooxygenase (COX) enzymes is the main mechanism that has been proposed to explain aspirin’s role in the development of cancers [6, 7].

The earliest clinical studies on the association between aspirin and cancer dates back to 1980; however these studies failed to establish the protective effect of aspirin on cancer incidence and mortality [8]. In 1988, a case-control study of 700 patients with colorectal cancer was the first to show that the use of aspirin was beneficial in reducing the risk of cancer [9]. Since then, an inverse association between the use of aspirin and the risk of developing cancer has been reported by many epidemiological studies [8–10]. However, not all of the data support the chemo-preventive effect of aspirin. Two large randomized controlled trials with 5-year and 10-year interventions, the Women’s Health Study and the Physicians’ Health Study, demonstrated no association between the use of aspirin and the incidence of any cancer [11, 12]. In addition, the CAPP2 investigators reported no protective effect of aspirin in a randomized clinical trial [13]. Moreover, the results of the Aspirin in Reducing Events in the Elderly trial showed an increased risk of cancer-related mortality in the aspirin group [14]. Furthermore, there is limited evidence on the association between aspirin use and the survival in patients with lung cancer (Table 1) [11, 15–22], and in addition, these studies revealed inconsistent results. In addition, the efficacy of aspirin use on the survival of lung cancer patients has to be validated in different racial and ethnic groups.

**Table 1:** Literatures relevant to the association of aspirin use and survival of patients with lung cancer

Source	Year	Country	Cell type	Stage	Study design	Study base	Sample size (users/non users)	HR (95%CI) for OS
Ratnasinghe et al. [15]	2004	USA	N/A	N/A	Prospective cohort	NHANES I&II	410 (178/232)	0.81 (0.62-1.07)
Cook et al. [11]	2005	USA	N/A	N/A	Randomized, double-blind, placebo-controlled	WHS	140 (58/82)	0.70 (0.50-0.99)
Fontaine et al. [16]	2010	UK	NSCLC	I-III	Prospective cohort	Hospital based	1765 (412/1353)	0.84 (N/A)
Brasky et al. [17]	2012	USA	N/A	N/A	Prospective cohort	VITamin And Lifestyle	434 (83/351)	0.99 (0.74–1.33)
Wang et al. [18]	2015	USA	NSCLC	III	Retrospective cohort	Hospital-based	673 (141/532)	0.97 (0.78–1.20)
McMenamin et al. [19]	2015	UK	N/A	N/A	Retrospective cohort	Population-based	13,388 (N/A)	1.00 (0.95–1.05)
Veitonmäki et al. [20]	2016	Finland	N/A	N/A	Retrospective cohort	FinRSPC	47 (3/44)	1.03(0.85–1.26)
Maddison et al. [21]	2017	UK	SCLC	N/A	Prospective cohort	Hospital-based	313 (71/242)	0.987 (0.754-1.293)
Kang et al. et al. [22]	2020	Korea	N/A	N/A	Retrospective cohort	Population-based	5938	1.03 (0.97-1.10)
Our work	2020	Taiwan	NSCLC	Inoperable	Retrospective cohort	Population-based (NHIRD)	38,842	0.79 (0.75–0.83)

*Abbreviations:* USA, United States of America; UK, United Kingdom; NSCLC, non-small cell lung cancer; SCLC, small cell lung cancer; N/A, not available; 95% CI, 95% confidence interval; NHANES, OS, Overall survival; National Health and Nutrition Examination Survey; HR, hazard ratio; FinRSPC, The Finnish Prostate Cancer Screening Trial; NHIRD, National Health Insurance Research Database

In this study, we investigated the effect of aspirin use on the survival of patients with inoperable NSCLC.

## Methods

### Ethics statement

The study protocol was approved by the Institutional Review Board of the Chang Gung Memorial Hospital at Chiayi (Chiayi, Taiwan) (No. 201901289B1). Since all the personal electronic data used in this study were analyzed anonymously in accordance with strict confidentially guidelines and regulations, the need for informed consent was waived by the Institutional Review Board.

Our research was performed in accordance with the ethical standards of the Declaration of Helsinki as revised in 1989. This study adhered to strict confidentiality guidelines that are in accordance with the regulations set by the Taiwan Personal Data Protection Act, as amended on May 26, 2010.

### Data source

We conducted a nationwide cohort study using population-based data from the Taiwan National Health Insurance Research Database (NHIRD). The National Health Insurance Program is a compulsory universal program established by the Taiwanese government. It has provided comprehensive health care for all the residents in Taiwan since March 1, 1995 and it currently covers approximately the entire population of 23.7 million people in this country. The NHIRD consists of the enrollment files, claims data, catastrophic illness files, and the registry for drug prescriptions. It represents one of the largest nationwide health care service databases in the world. The diagnostic accuracy of the NHIRD has been validated previously for major diseases [23], and its clinical consistency in cancer research had been proved [24].

The Registry for Catastrophic Illness Patient Database (RCIPD) is a subset of the NHIRD. It is a registry for severe illnesses, including cancer, cirrhosis, and autoimmune diseases. All the applications for catastrophic illness certification are reviewed rigorously by experts. At least two independent clinical physicians review the medical records and laboratory, histological, and imaging data of each patient with malignancy who applies for catastrophic illness certification. Therefore, the diagnosis of cancer can be considered to be accurate [25].

### Study cohort

We conducted a population-based retrospective cohort study using the NHIRD between January 1, 2000 and December 31, 2012. Lung cancer was defined according to the International Classification of Disease, Ninth Revision, Clinical Modification code (ICD-9-CM code 162) from the RCIPD (*n* = 104,963). The index date was defined as the date of the first medical visit with an ICD-9 CM code for lung cancer.

**Fig. 1:**
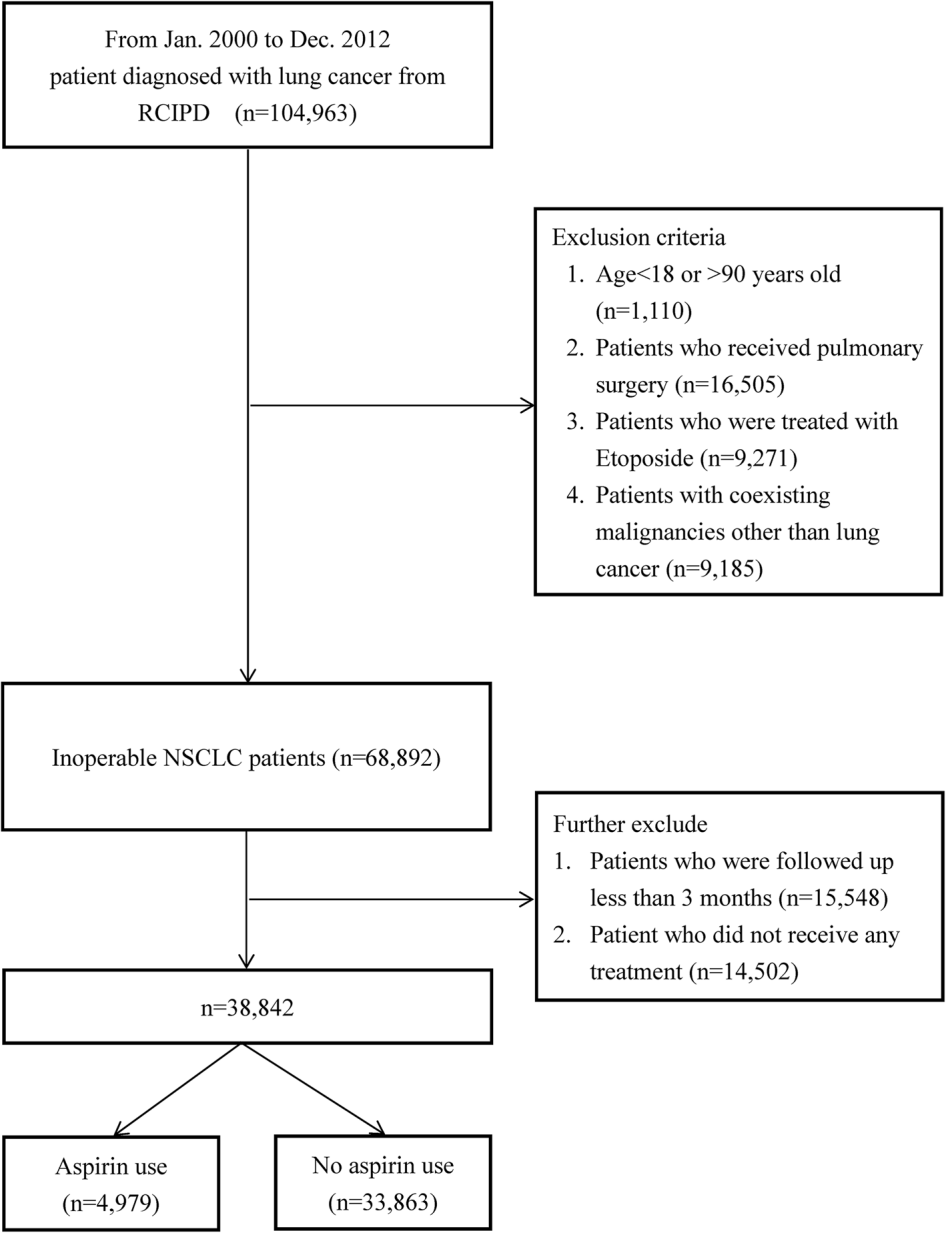
Flow chart of patient enrollment process of the study cohort. ICD-9-CM, International Classification of Diseases, Ninth Revision, Clinical Modification; NSCLC, non-small cell lung cancer

Patients < 18 or > 90 years of age (*n* = 1110) were excluded. Patients with co-existing malignancies other than lung cancer (*n* = 9185) were also excluded. Although a histopathologic confirmation was required for the issuing of a catastrophic illness certificate of lung cancer, information on cell type and the clinical stage of lung cancer was not available in the RCIPD. Patients treated with etoposide (*n* = 9271) were suspected of having small cell lung cancer and were therefore excluded from this study. Operable lung cancer patients (*n* = 16,505) were defined as those having insurance claims for pulmonary surgeries, including wedge resections, segmentectomies, lobectomies, and pneumonectomies, and these were also excluded from this study. Patients who did not receive any treatment (*n* = 14,502) and patients with a follow-up time of < 3 months (*n* = 15,548) were also excluded, leaving 38,842 patients in the final analysis (Fig. 1).

Each patient was followed up until 6 years after the index date, death, or the end of 2012. The median follow-up time for the aspirin users was 1.73 (interquartile range [IQR] 0.94–3.53) years and the median follow-up time for the non-aspirin users was 1.30 (IQR 0.69–2.62) years.

Death was defined as the withdrawal of the patient from the NHI program. This definition was used in a previous study [26]. Aspirin users were defined as those who used aspirin for > 28 defined daily doses (DDD) after the NSCLC diagnosis.

### Matched cohort

To confirm the association of aspirin use and NSCLC survival, a propensity score analysis was used to reduce the possible confounding factors such as the Charlson Comorbidity Index (CCI), sociodemographic characteristics (age, sex, income, and level of urbanization), and lung cancer treatments.

A propensity score matching procedure was performed using a multivariable logistic model with a greedy algorithm based on 8-digit to 1-digit matching with no replacements. Aspirin users and non-users were matched at a ratio of 1:1. In total, 9864 insured adults (4932 matched pairs) were included in the matched cohort.

The median follow-up time for the aspirin users was 1.73 (IQR 0.94–3.53) years and the median follow-up time for the non-aspirin users was 1.22 (IQR 0.66–2.34) years.

### Primary study outcome

Overall survival (OS) was considered as the primary outcome. It was calculated as the time interval between the index date of the NSCLC diagnosis and the date of death (defined as the date of withdrawal from the insurance system).

### Statistical analyses

While both time-dependent Cox regression models and landmark analyses are useful in resolving the problem of immortal time bias, the time-dependent Cox regression is the most appropriate method for analyzing cumulative and long-term drug exposure [27], and this method was used in a previous study [19].

In this study, Cox proportional hazard models with time-dependent covariates were used to estimate the hazard ratios (HRs) and the accompanying 95.0% CIs after the adjustment for the CCIs, the sociodemographic characteristics (age, sex, income, and level of urbanization), and lung cancer treatments.

The outcomes of different patient groups, stratified according to sex, age, the CCI, and lung cancer treatments, were also analyzed. All the analyses were conducted using SAS statistical software (Version 9.4; SAS Institute, Cary, NC, USA). A two-tailed *P* < 0.05 was considered to be statistically significant.

## Results

A total of 38,842 patients diagnosed with inoperable NSCLC from 2000 to 2012 were included in our study. Of these patients, 4979 patients used aspirin after the NSCLC diagnosis. The median time of aspirin use was 0.47 (IQR, 0.12–1.16) years, with a mean of 0.88 ± 1.12 years.

In the study cohort, aspirin users were older (*P* < 0.001) and had a significantly higher proportion were male. Aspirin users were also more likely to have comorbidities (*P* < 0.001) than non-aspirin users. All the covariates were well balanced after adjusting for the propensity scores (Table 2). The median dose of aspirin was 102.38 ± 59.64 DDD in the study group and 102.41 ± 59.93 DDD in the matched group.

**Table 2:** Demographic and clinical characteristics of patients in the study (*n* = 38,842) and matched (*n* = 9864) cohorts

Characteristic	Study Cohort				Matched Cohort			
	Aspirin (***n*** = 4979)	No Aspirin (***n*** = 33,863)	***P-***value	Standardized difference	Aspirin (***n*** = 4932)	No Aspirin (n = 4932)	***P***-value	Standardized difference
Age, years (mean ± SD)	71.48 ± 9.51	64.93 ± 12.21	< 0.0001^*^	0.599	71.41 ± 9.50	71.93 ± 9.55	0.065	0.055
Age			< 0.0001^*^				0.0913	
< 65, n (%)	1060 (21.29)	15,191 (44.86)		0.517	1057 (21.43)	989 (20.05)		0.034
≥65, n (%)	3919 (78.71)	18,672 (55.14)		0.517	3875 (78.57)	3943 (79.95)		0.034
Sex, n (%)			< 0.0001^*^				0.28	
Male	3245 (65.17)	20,536 (60.64)		0.094	3205 (64.98)	3256 (66.02)		0.022
Female	1734 (34.83)	13,327 (39.36)		0.094	1727 (35.02)	1676 (33.98)		0.022
Income (NTD), n (%)			< 0.0001^*^				0.2085	
0 (Dependent)	1036 (20.81)	5841 (17.25)		0.091	1024 (20.76)	1074 (21.78)		0.025
1–15,840	991 (19.90)	5476 (16.17)		0.097	978 (19.83)	1009 (20.46)		0.016
15,841–25,000	2226 (44.71)	16,673 (49.24)		0.091	2209 (44.79)	2190 (44.40)		0.008
≥25,000	726 (14.58)	5873 (17.34)		0.075	721 (14.62)	659 (13.36)		0.036
Urbanization, n (%)			0.5737				0.5648	
1 (City)	1380 (27.72)	9088 (26.84)		0.020	1367 (27.72)	1354 (27.45)		0.006
2	2141 (43.00)	14,758 (43.58)		0.012	2120 (42.98)	2124 (43.07)		0.002
3	912 (18.32)	6193 (18.29)		0.001	906 (18.37)	875 (17.74)		0.016
4 (Village)	546 (10.97)	3824 (11.29)		0.010	539 (10.93)	579 (11.74)		0..026
CCI, n (%)			< 0.0001^*^				0.3988	
≤6	2994 (60.13)	17,441 (51.50)		0.174	2955 (59.91)	2996 (60.75)		0.017
> 6	1985 (39.87)	16,422 (48.50)		0.174	1977 (40.09)	1936 (39.25)		0.017
Comorbidities, n (%)								
Acute myocardial infarction	339 (6.81)	314 (0.93)	< 0.0001^*^	0.309	295 (5.98)	249 (5.05)	0.0425	0.041
Ischemic cerebrovascular accident	138 (2.77)	488 (1.44)	< 0.0001^*^	0.903	134 (2.72)	127 (2.58)	0.6606	0.009
Chronic kidney disease	293 (5.88)	932 (2.75)	< 0.0001^*^	0.155	285 (5.78)	274 (5.56)	0.6319	0.010
Diabetes mellitus	1851 (37.18)	6507 (19.22)	< 0.0001^*^	0.407	1825 (37.00)	1802 (36.54)	0.6310	0.010
Hypertension	3954 (79.41)	15,041 (44.42)	< 0.0001*	0.773	3907 (79.22)	3979 (80.68)	0.0702	0.036
Dyslipidemia	2289 (45.97)	7666 (22.64)	< 0.0001^*^	0.507	2248 (45.58)	2170 (44.00)	0.1143	0.032
Atrial fibrillation	380 (7.63)	696 (2.06)	< 0.0001*	0.262	372 (7.54)	335 (6.79)	0.1487	0.029
GI bleeding	228 (4.58)	1067 (3.15)	< 0.0001*	0.074	225 (4.56)	211 (4.28)	0.4928	0.014
Lung cancer treatment, n (%)								
Chemotherapy	4057 (81.48)	28,723 (84.82)	< 0.0001^*^	0.089	4027 (81.65)	4033 (81.77)	0.8758	
Erlotinib	720 (14.46)	4637 (13.69)	0.1426	0.022	713 (14.46)	646 (13.10)	0.0503	0.039
Gefitinib	1034 (20.77)	6351 (18.75)	0.0007^*^	0.051	1018 (20.64)	943 (19.12)	0.0585	0.038
Radiotherapy	2575 (51.72)	18,446 (54.47)	0.0003^*^	0.055	2554 (51.78)	2539 (51.48)	0.7625	0.006

Statistical significance is define by *P*<0.05. *P* values were marked "*" if they were below this threshold

**Fig. 2:**
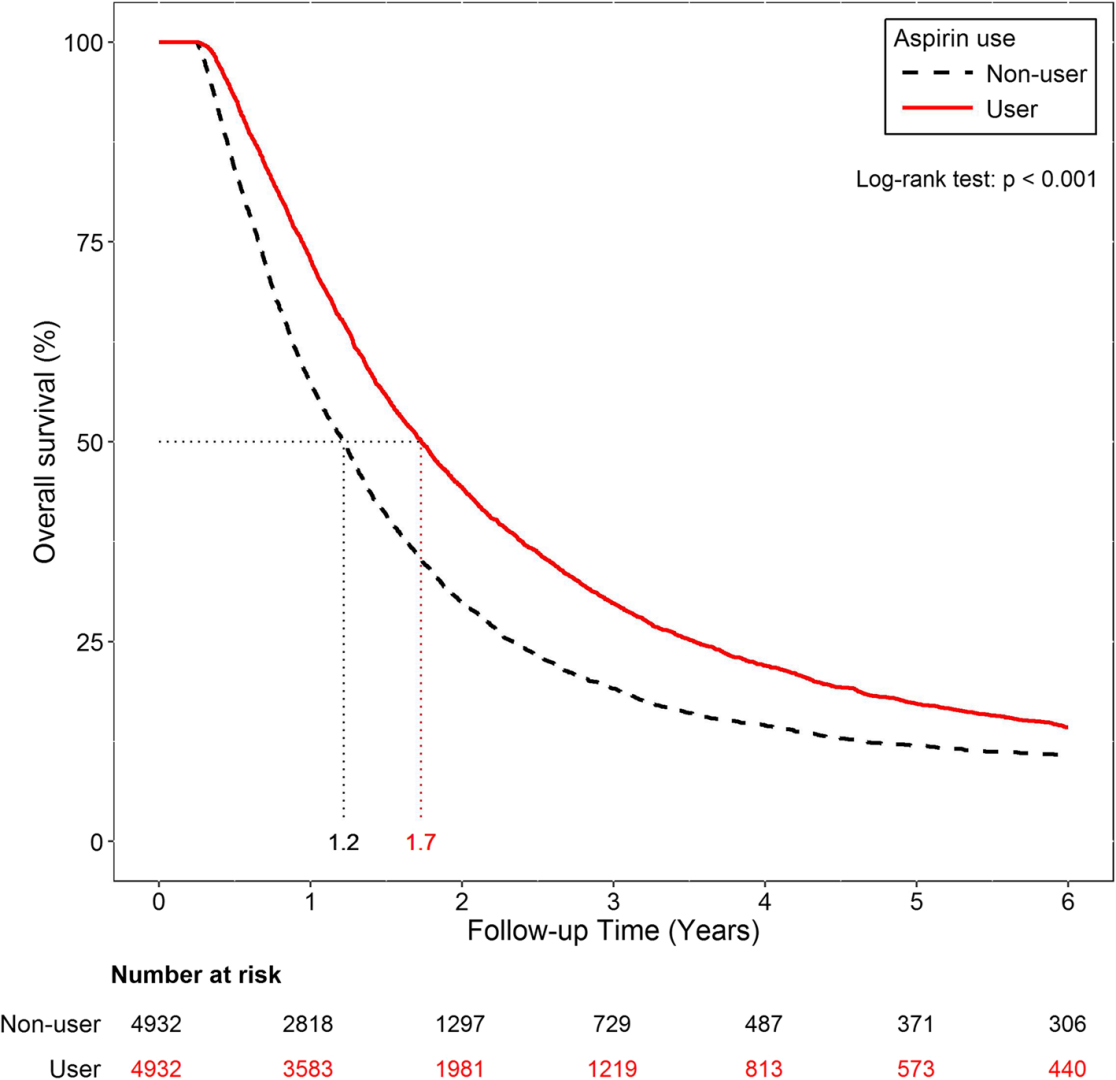
Kaplan-Meier curves of overall survival for patients with or without aspirin in the matched cohort. Aspirin use was associated with a significantly longer overall survival

Survival analysis was also performed for patients treated with or without aspirin in the matched cohort using Kaplan-Meier curves. The median OS of the aspirin users was 1.73 (IQR, 0.94–3.53) years compared with 1.30 (IQR, 0.69–2.62) years of the non-aspirin users (Fig. 2).

**Fig. 3:**
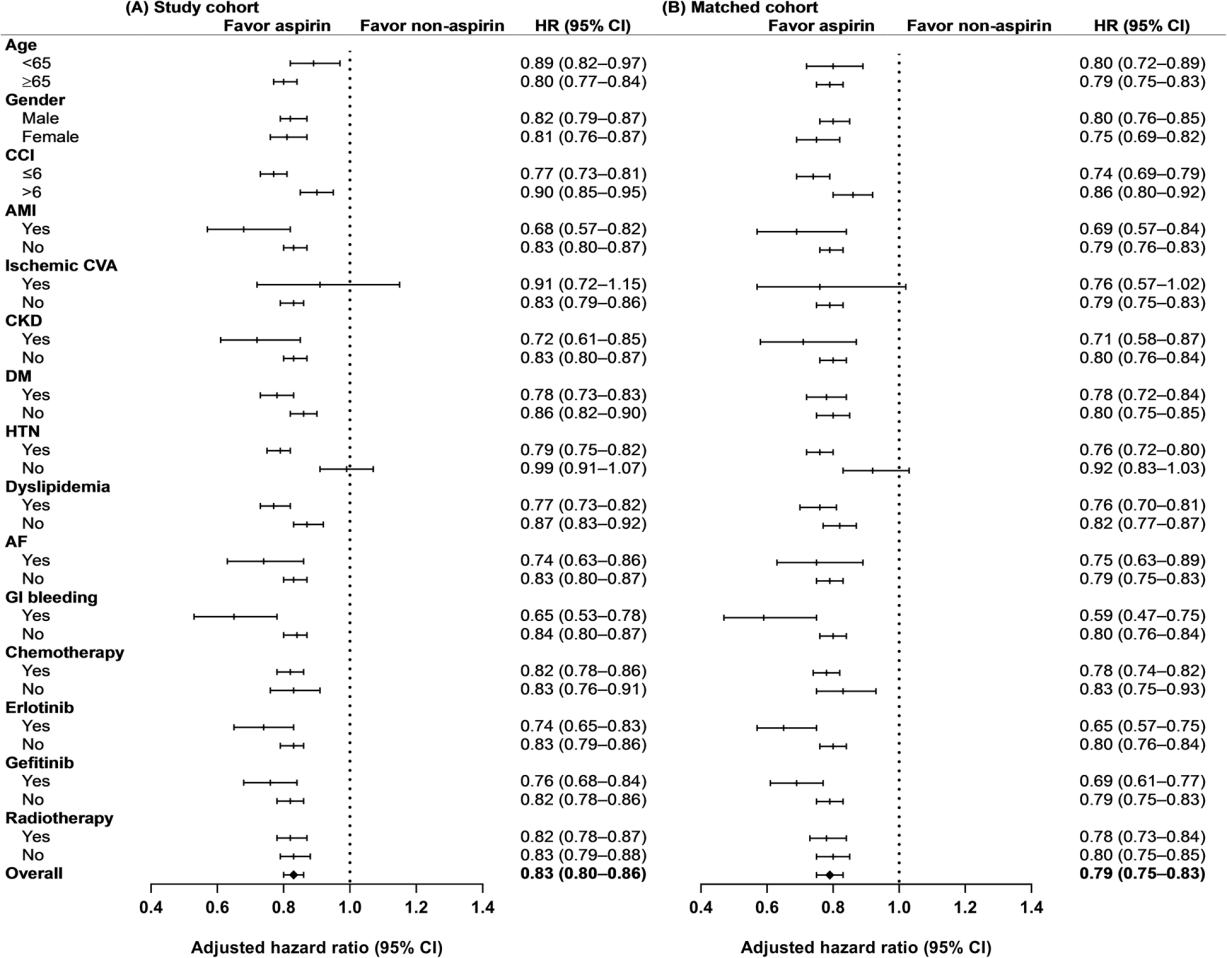
Subgroup analysis of adjusted hazard ratios (HRs) of risk factors for aspirin-related mortality. Aspirin use was associated with a significantly longer OS (HR: 0.83, 95.0% CI: 0.80–0.86). The survival benefit of aspirin use was maintained after propensity score matching at a ratio of 1:1 (HR: 0.79, 95.0% CI: 0.75–0.83)

The Cox proportional hazard model with the time-dependent covariate showed that aspirin use was associated with a significantly longer OS (HR: 0.83, 95.0% CI: 0.80–0.86). The survival benefit of aspirin use was maintained after propensity score matching at a ratio of 1:1 (HR: 0.79, 95.0% CI: 0.75–0.83) (Fig. 3).

The subgroup analysis stratified by age, sex, and the CCI also revealed a significant survival benefit for the aspirin users except for those who experienced ischemic cerebrovascular accidents (HR: 0.91, 95.0% CI: 0.72–1.15), and those without hypertension (HR: 0.99, 95.0% CI: 0.91–1.07). The survival benefit of aspirin use remained significant when the subgroup analysis was repeated by propensity score matching except for those who experienced ischemic cerebrovascular accidents (HR: 0.76, 95.0% CI: 0.57–1.02), and those without hypertension (HR: 0.92, 95.0% CI: 0.83–1.03) (Fig. 3).

## Discussion

In this retrospective, nationwide, population-based cohort study, we observed that aspirin use was associated with a longer OS in patients with inoperable NSCLC. This finding offered further evidence of the potential anti-tumorigenic effects of aspirin.

While aspirin was extensively used for its analgesic, antipyretic and anti-inflammatory properties, it was not until 1971 that its exact mechanism of action was elucidated by John Vane who showed that aspirin decreased the production of prostaglandin (PG) by inhibiting a rate-limiting enzyme named COX [28]. The discovery of its anti-platelet effects led to the increasing use of aspirin from the 1980s, in the prevention of both myocardial infarction and stroke [28, 29].

While a case-control study of 700 patients with colorectal cancer conducted in Melbourne, Australia was the first to show a possible anti-cancer effect in human cancer [9], randomized controlled trials showed no association between aspirin use and cancer incidence [11, 30, 31]. However, these studies were designed to examine the effect of aspirin on vascular disease, and the effects of aspirin on cancer were not the primary endpoint.

Nonetheless, further studies established the chemo-preventive effect of aspirin in colorectal cancer [32, 33] and these inspired numerous studies on the potential preventive role of aspirin on other cancers [34–36], contributing to the publication of abundant systematic reviews and meta-analyses [37–39].

The studies focused more on the role of aspirin in lung cancer incidence [8–14, 39] and despite promising in vivo experimental data [40], few attempts were made to study the association between the use of aspirin and lung cancer survival (Table 1). The findings in these studies were inconsistent and many studies did not reflect the time-dependent anticancer effects of the exposure of aspirin on lung cancer, appropriately. Three studies provided limited information since they were not population-based [16, 18, 21], while four studies used a cohort design to investigate other issues. Aspirin use on lung cancer survival was not the primary endpoint of these trials [11, 15, 17, 20].

A meta-analysis of three randomized trials that were originally conducted for the prevention of vascular events, reported that a comparison of aspirin versus a placebo showed that aspirin was protective with regard to lung mortality (HR: 0.71, 95.0% CI: 0.58–0.89) [41], which is consistent with the result of our study, which showed that aspirin use is associated with an improved OS in inoperable NSCLC patients. Using a big data analysis approach, our study provided further evidence in support of the potential antineoplastic effect of aspirin.

The first experimental animal models that proposed that aspirin could be of benefit against cancer were developed more than half a century ago, when Gasic and colleagues observed that thrombocytopenia in tumor-bearing mice was associated with a 50% reduction in lung metastases [42, 43].

Notably, the anti-neoplastic effect of aspirin was mediated through its inhibition of COX enzymes that promote carcinogeneis through the synthesis of PG. [44] Apart from inhibiting the synthesis of PG, aspirin has also been shown to upregulate tumor-suppression genes and inhibit NF-kB activation, thus illustrating its anticancer activities in a COX independent pathway [45, 46]. On the other hand, mounting preclinical evidence suggests that aspirin may exhibit anti-neoplastic effects by inducing apoptosis [47, 48] suppressing angiogenesis [49], and inhibiting the proliferation of tumor cells [50].

There were several strengths that need to be emphasized in the present study. First, with the use of a nationwide population-based health insurance claims database, our study cohort may represent patients with NSCLC in real world. Second, the study cohort was retrieved from a computerized database comprising all NSCLC patients diagnosed between January 1, 2000 and December 31, 2013, reducing the potential for recall and selection bias. Third, the results of our study were validated using an alternative statistical method. After matching aspirin users and non-users at a ratio of 1:1, according to confounding factors such as the sociodemographic characteristics, CCI, and lung cancer treatments, the results were found to be comparable between the two approaches.

Nevertheless, certain limitations of our study should be considered. First, the major limitation of our study was that information about the histology and the TNM staging of the NSCLC patients was not available in the NHIRD. In addition, several unmeasured confounding factors, including smoking histories, body mass indexes, family histories and presence of environmental toxins, which are associated with OS, were not included in the database. For this reason, we used a cohort of inoperable NSCLC patients who can be regarded as having advanced-stage disease. We considered that most of these patients died because of cancer progression and that the potential effect of confounding factors of OS should be marginal.

Second, given the natural of this retrospective study based on diagnostic codes and pharmacy claim records, heterogeneity existed between the aspirin users and non-aspirin users. In order to deal with this limitation, propensity score matching was used to adjust for the potential confounding factors and a significant survival benefit still existed in the aspirin group. Third, we assumed that all the prescribed aspirin were taken by the patients. However, this could have resulted in an overestimation of the actual dosage due to a lack of adherence. Fourth, the dose-response relationship was not evaluated in our study and this could have decreased the impact of our study. Fifth, detailed information on chemotherapy and radiotherapy were not available in the NHIRD database and could not be included in the analysis. Therefore, caution is necessary when interpreting the study findings.

Finally, we defined aspirin users as those who use aspirin for > 28 defined daily doses after the NSCLC diagnosis. Immortal time bias may play a role and the protective effects of aspirin may be an artifact of immortal time bias. Therefore, although we used the time-dependent Cox regression for further analysis (yearly aspirin use < 28 DDD as a reference group) to reduce the effect of immortal time bias, the results still showed a survival benefit in aspirin users.

## Conclusions

The results of our study suggest that among inoperable NSCLC patients, aspirin use is associated with an improved OS. Despite the need for future prospective randomized clinical trials, aspirin may be considered as an additional treatment for inoperable NSCLC patients.

## Data Availability

Data are available from the National Health Insurance Research Database (NHIRD) published by Taiwan National Health Insurance (NHI) Bureau. Due to legal restrictions imposed by the government of Taiwan in relation to the “Personal Information Protection Act”, data cannot be made publicly available. Requests for data can be sent as a formal proposal to the NHIRD (http://nhird.nhri.org.tw).
